# Assessing glomerular filtration rate in patients with severe heart failure: comparison between creatinine-based formulas

**DOI:** 10.1590/S1516-31802012000500004

**Published:** 2012-11-13

**Authors:** Alexandre Libório, Russian Uchoa, João Neto, Juan Valdivia, Elizabeth De Francesco Daher, Juan Mejia

**Affiliations:** I MD, PhD. Professor, Postgraduate Program on Public Health, Universidade de Fortaleza (Unifor), Fortaleza, Ceará, Brazil.; II Medical student. Universidade de Fortaleza (Unifor), Fortaleza, Ceará, Brazil.; III MD. Cardiologist, Cardiac Transplantation Service, Hospital Messejana, Fortaleza, Ceará, Brazil.; IV Medical Student. Universidade de Fortaleza (Unifor), Fortaleza, Ceará, Brazil.; V MD, PhD. Professor, Department of Clinical Medicine, Universidade Federal do Ceará (UFC), Fortaleza, Ceará, Brazil.; VI MD. Coordinator of the Heart Transplantation Program, Hospital Messejana, Fortaleza, Ceará, Brazil.

**Keywords:** Heart failure, Glomerular filtration rate, Kidney failure, chronic, Heart transplantation, Renal insufficiency, Insuficiência cardíaca, Taxa de filtração glomerular, Falência renal crônica, Transplante de coração, Insuficiência renal

## Abstract

**CONTEXT AND OBJECTIVE:**

Severe heart failure is highly associated with chronic kidney disease (CKD). Serum creatinine is a poor indicator of renal function and glomerular filtration rate (GFR) estimation is an accessible method for assessing renal function. The most popular formulas for GFR estimation are the Cockcroft-Gault (CG), the four-variable Simplified Modification of Diet in Renal Disease (sMDRD) and the recently introduced CKD-Epidemiology Collaboration (CKD-EPI). The objective of the study was to analyze the correlation between these three equations for estimating GFR in patients with severe heart failure.

**DESIGN AND SETTING:**

Cross-sectional observational study at a university reference center.

**METHODS:**

GFR was estimated in patients with severe heart failure who were awaiting heart transplantation, using the CG, sMDRD and CKD-EPI formulas. These estimates were analyzed using Pearson’s correlation and Bland-Altman analysis.

**RESULTS:**

This study included 157 patients, of whom 32 (20.3%) were female. Normal serum creatinine concentration was observed in 21.6%. The mean GFR according to CG, sMDRD and CKD-EPI was 70.1 ± 29.5, 70.7 ± 37.5 and 73.7 ± 30.1 ml/min/1.73 m^2^; P ≥ 0.05. Pearson’s coefficient demonstrated good correlations between all the formulas, as did Bland-Altman. However, the patients presented GFR < 60 ml/min more frequently with the sMDRD formula (54.1% versus 40.2% for CG and 43.2% for CKD-EPI; P = 0.02).

**CONCLUSION:**

Despite the good correlation and agreement between the three methods, the sMDRD formula classified more patients as presenting GFR less than 60 ml/min.

## RESUMO

**CONTEXTO E OBJETIVO:**

Insuficiência cardíaca grave tem elevada correlação com doença renal crônica. Creatinina sérica é um indicador pobre da função renal e a estimativa da taxa de filtração glomerular (TFG) é um método acessível para avaliar a função renal. As fórmulas mais populares estimadoras da TFG são a Cockcroft-Gault (CG), a modificação de dieta simplificada em doença renal (Simplified Modification of Diet in Renal Disease, sMDRD) com quatro variáveis, e a recentemente introduzida Colaboração Epidemiológica na Doença Renal Crônica (Chronic Kidney Disease-Epidemiology Collaboration, CKD-EPI). O objetivo foi analisar a correlação entre essas três equações estimadoras da TFG em pacientes com insuficiência cardíaca grave.

**TIPO DE ESTUDO E LOCAL:**

Estudo transversal observacional em centro de referência universitário.

**MÉTODOS:**

Pacientes com insuficiência cardíaca grave em fila para transplante cardíaco tiveram a TFG estimada pelo CG, sMDRD e CKD-EPI. Essas estimativas foram analisadas pela correlação de Pearson e análise de Bland-Altman.

**RESULTADOS:**

Foram incluídos 157 pacientes: 32 (20,3%) mulheres. Creatinina sérica normal foi observada em 21,6%. TFG média de acordo com CG, sMDRD e CKD-EPI foi 70,1 ± 29,5, 70,7 ± 37,5 e 73,7 ± 30,1 ml/min/1.73 m^2^; P ≥ 0,05. Coeficiente de Pearson mostrou boa correlação entre todas as fórmulas, assim como Bland-Altman. Entretanto, os pacientes apresentaram TFG < 60 ml/min mais frequentemente com a fórmula sMDRD (54,1% *versus* 40,2% para CG e 43,2% para CKD-EPI; P = 0,02).

**CONCLUSÃO:**

Apesar da boa correlação e concordância entre os três métodos, a fórmula do sMDRD classificou mais pacientes com TFG menor que 60 ml/min.

## INTRODUCTION

Renal dysfunction is highly prevalent in patients with heart disease, mainly as a result of concomitant diabetes mellitus, hypertension or congestive heart failure.^1^ Moreover, development of chronic kidney disease (CKD), i.e. a glomerular filtration rate (GFR) < 60 ml/min, is predictive of premature cardiovascular death.^2^ Heart transplantation is the definitive treatment for eligible patients with end-stage heart failure, but the immunosuppressive therapy that is required, especially calcineurin inhibitors, represents an additional risk factor for renal failure.^3^^,^^4^

Regimens containing calcineurin inhibitors are not used in individuals with severe renal impairment, in order to avoid additional drug-induced nephrotoxicity. Thus, GFR monitoring is an important tool in managing heart failure patients, both before and after heart transplantation.

Serum creatinine is a poor indicator of renal function, and GFR estimation is preferred in assessing renal function.^5^ The formulas for GFR estimation typically include age and gender, in order to accommodate differences in creatinine generation. The most popular formulas include Cockcroft-Gault (CG) and the four-variable Simplified Modification of Diet in Renal Disease (sMDRD).^4^^,^^6^^,^^7^ Over recent years, this simplified formula has been introduced into clinical practice, and it is currently considered to be the best available formula.^8^ Generalization of these formulas to specific populations (e.g. heart failure or liver disease patients) is troublesome, mainly because of poor nutritional status, low muscle mass, edema and weight loss. Recently, a new equation, the Chronic Kidney Disease-Epidemiology Collaboration (CKD-EPI), was proposed for estimating GFR. There have been claims that it is as accurate as sMDRD for diagnosing cases of GFR less than 60 ml/min and that its performance among patients with GFR greater than 60 ml/min is better.^9^ However, no study has compared the CKD-EPI, CG and sMDRD equations in a specific population with severe heart failure awaiting orthotopic heart transplantation.

## OBJECTIVE

The aim of this study was to analyze the correlations between creatinine-based equations for estimating GFR, among patients with severe heart failure who were awaiting heart transplantation.

## METHODS

This was a correlation study that included 157 consecutive patients who underwent orthotopic heart transplantation in a tertiary center in northeastern Brazil between 2004 and 2010. Patients under 18 years of age and those who had needed hospitalization during the preceding month were excluded. After recruitment for heart transplantation, demographic data and venous blood samples were obtained. Serum creatinine was measured by using a kinetic alkaline picrate assay validated against isotope dilution mass spectrometry (IDMS).

The estimated GFR was obtained through three methods (equations):

(1) sMDRD equation: GFR (expressed in ml/min/1.73 m^2^) = 186 x [cr] ^-1.154^ x [age] ^-0.203^ x [0.742 if patient was female];

(2) CG formula normalized to a body surface area of 1.73 m^2^, with creatinine clearance expressed in ml/min/1.73 m^2^: GFR (males) = 1.23 x weight (kg) x [140-age]/plasma creatinine (mmol/l) x 1.73/BSA; GFR (females) = 1.03 x weight (kg) x [140-age]/plasma creatinine (mmol/l) x 1.73/BSA, where BSA (m^2^) = [weight (kg) x height (cm)/3600];

(3) CKD-EPI formula using the following equations:

For women with creatinine < 0.7 mg/dl (62 mmol): GFR = 144 x (cr/0.7)-0.329 x (0.993) x age.
For women with creatinine > 0.7 mg/dl (62 mmol): GFR = 144 × (cr/0.7)-1.209 x (0.993) x age.
For men with creatinine < 0.9 mg/dl (80 mmol): GFR = 141 x (cr/0.9)-0.411 x (0.993) x age.
For men with creatinine > 0.9 mg/dl (80 mmol): GFR = 141 × (cr/0.9)-1.209 x (0.993) x age.

All the patients were considered to be non-black because of the special miscegenation of the Brazilian population. The data were expressed as the mean ± standard deviation (SD). The unpaired t test or one-way analysis of variance (ANOVA) were used to compare means between pairs of groups or more than two groups, respectively. The chi-square test was used for categorical variables. Pearson’s correlation coefficient was obtained using log-transformed data. The means of the absolute differences between pairs of methods were obtained. Bland-Altman plots were constructed to illustrate the degree of agreement between each prediction equation and the measured GFR. GraphPad Prism version 5.0 was used for the statistical analysis.^3^

## RESULTS

**Figure 1.**
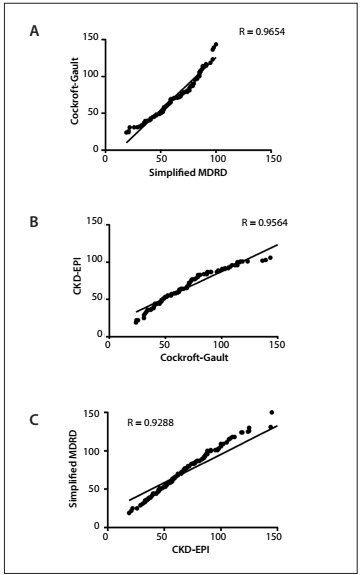
Pearson’s correlation using: (A) Cockcroft-Gault and simplified Modification of Diet in Renal Disease (sMDRD), (B) Cockcroft-Gault and Chronic Kidney Disease-Epidemiology Collaboration (CKD-EPI) equation and (C) simplified MDRD and CKD-EPI.

**Figure 2.**
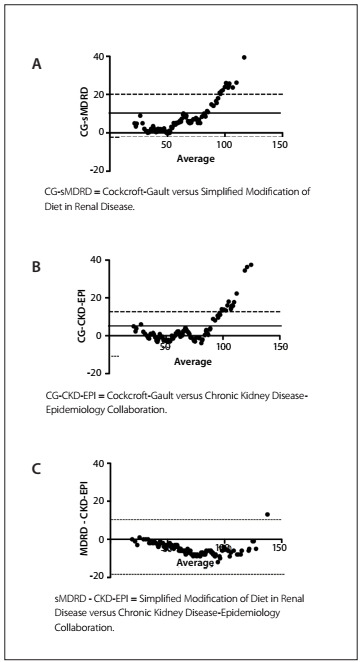
(A, B and C) Bland-Altman plots comparing each of the prediction equations studied.

A total of 157 patients, of whom 32 (20.3%) were females, were included in this study. The main indications for orthotopic heart transplantation were ischemic cardiomyopathy (47%), dilated cardiomyopathy (39%) and Chagas cardiomyopathy (23%). The patients’ mean age was 47.5 ± 14 years (males 48 ± 13.8 and females 45.2 ± 16.8; P = 0.74 not significant. The mean serum creatinine immediately before orthotopic heart transplantation was 1.22 ± 0.51 mg/dl (males 1.24 ± 0.52 and female 1.0 ± 0.28 mg/dl; P = 0.04). Normal serum creatinine concentration (i.e. less than 1.5 mg/dl in males and 1.2 mg/dl in females) was observed in 21.6% of the patients (males 22.4% and females 18.7%; P = not significant). The mean GFR according to CG, sMDRD and CKD-EPI was 70.1 ± 29.5, 70.7 ± 37.5 and 73.7 ± 30.1 ml/min/1.73 m^2^; P = not significant. Pearson’s coefficient demonstrated good correlations between all the predictive formulas, as can be seen in Figure 1. Comparison of the GFR findings using the Bland-Altman method showed that the level of agreement between the methods was significant, especially between sMDRD and CKD-EPI, across the entire mean spectrum. Analysis on the graphs using the CG formula (Figures 2a and b) showed that there was greater disagreement when the GFR was greater than 70 to 80 ml/min. There was greater bias between the CG and sMDRD formulas (Table 1).

**Table 1.** Estimated glomerular filtration rate (GFR) using the Cockcroft-Gault (CG), simplified Modification of Diet in Renal Disease (sMDRD) and Chronic Kidney Disease-Epidemiology Collaboration (CKD-EPI) formulas

	Mean absolute difference ? SD	Median absolute difference
CG versus sMDRD	9.06 ? 11.53	5.59
CG versus CKD-EPI	4.02 ? 10.05	0.81
sMDRD versus CKD-EPI	-2.98 ? 14.74	-5.00

SD = standard deviation.

Although there were good correlations and low bias between these creatinine-based formulas, it was seen that when the patients were distributed according GFR levels as presented in Table 2, the sMDRD equation was significantly more sensitive for classifying patients with GFR < 60 ml/min, i.e. 54.1% versus 40.2% for CG and 43.2% for CKD-EPI; P = 0.02 from the chi-square test. Moreover, disagreement occurred in 40 (25.5%) of the patients when sMDRD and CKD-EPI were compared, and in 47 (30%) when sMDRD and CG were compared. In contrast, only 18 patients (11.46%) were in different stages when CG and CKD-EPI were compared.^4^

**Table 2.** Distribution of patients according to chronic kidney disease (CKD) stages

GFR	Cockcroft-Gault	Simplified MDRD	CKD-EPI
> 90 ml/min	32 (20.3%)	13 (8.3%)	27 (17.2%)
60-89 ml/min	62 (39.5%)	59 (37.6%)	62 (39.6%)
45-59 ml/min	29 (18.5%)	49 (31.2%)	34 (21.5%)
30-44 ml/min	29 (18.5%)	27 (17.1%)	25 (15.9%)
15-29 ml/min	5 (3.2%)	9 (5.8%)	9 (5.8%)

MDRD = Modification of Diet in Renal Disease; CKD-EPI = Chronic Kidney Disease-Epidemiology Collaboration.

## DISCUSSION

To the best of our knowledge, this is the first study evaluating the new CKD-EPI equation among patients with severe heart failure who were awaiting heart transplantation. We found acceptable agreement in comparing the CKD-EPI values with both the CG and the sMDRD equations, but sMDRD was more sensitive for classifying patients with GFR < 60 ml/min.

Kidney disease affects cardiac performance through electrolyte imbalance, volume overload and negative inotropy.^10^ In a retrospective analysis within the Studies of Left Ventricular Dysfunction (SOLVD) trial, even moderate degrees of renal insufficiency, as measured using the CG equation, were independently associated with an increased risk for all-cause mortality in patients with heart failure.^11^ Correct identification and classification of renal failure in patients with advanced heart failure also has central importance in choosing immunosuppressive therapy, especially regarding decisions about calcineurin inhibitors. MDRD was proposed by Levey et al.^6^ and, since then, many studies have demonstrated its accuracy for estimating GFR. In patients with advanced heart failure, MDRD was found to present better performance than shown by CG, for predicting GFR less than 60 ml/min, using 51Cr-EDTA measurements as a reference.^12^ In the same study, the simplified MDRD formula had a mean bias of only 10.9 ml/min, compared with 51Cr-EDTA. In our data, the greatest mean bias between the methods was found between the CG and sMDRD formulas and was similar to the previous finding (9.06 ml/min).

The CKD-EPI equation was proposed recently as a more accurate method for estimating GFR than MDRD.^9^ Even among patients with GFR above 60 ml/min, for which MDRD has poor performance, CKD-EPI has been demonstrated to be more accurate in other studies. In patients with advanced heart failure, we demonstrated that the mean bias difference between MDRD and CKD-EPI was only -2.98 ml/min. Moreover, almost all the points are within the agreement limits (Figure 2c). With regard to special populations, CKD-EPI has been used among diabetics,^13^ preeclamptic women,^14^ elderly patients,^15^ candidates for liver transplantation^16^ and individuals who have undergone orthotopic heart transplantation.^17^ The National Kidney Foundation^5^ has created an operational definition and classification of chronic kidney disease stages that provides estimates of disease prevalence according to stage, thus making it possible to develop a broad overview for a “clinical action plan” to evaluate and manage each stage of chronic kidney disease, and to define the individuals who are at greater risk of developing chronic kidney disease. This classification is largely based on the GFR.

Although our patients could not be diagnosed as having CKD because they did not have a second GFR measurement after an interval of least three months, precise staging is needed in order to correctly manage these patients. Hence, accuracy of GFR measurements becomes an important endpoint when analyzing different methods for estimating GFR.

Among heart transplant recipients, the prevalence of CKD is high and probably underappreciated.^18^ Malyszko et al.^17^ used the MDRD formula and found that 63% of the patients had GFR less than 60 ml/min after heart transplantation. The results were similar when CKD-EPI was used. In our data, there was no agreement between GFR formulas in determining renal failure prevalence: there was a considerable difference in allocating patients with significant renal failure (GFR < 60 ml/min): 54.1% from sMDRD and only 43.2% from CKD-EPI (P < 0.05). This finding may have important implications with regard to selecting the appropriate strategy for individually tailored therapy in order to achieve the best possible outcomes in relation to renal function after transplantation.

While the MDRD formula is a good method for estimating GFR, it has not been a useful tool in predicting outcomes among patients with heart failure. Gardner et al. reported that N-terminal prohormone brain natriuretic peptide (NT-proBNP) was a better prognostic marker than GFR from MDRD, among patients with advanced chronic heart failure.^19^ Scrutinio et al.^20^ studied a population with normal serum creatinine and also demonstrated that the CG equation was better than MDRD for predicting heart failure-associated outcomes. The question of whether the greater sensitivity of MDRD for classifying advanced heart failure patients with renal failure reflects progressive decline in renal function or greater mortality among this population after heart transplantation remains to be addressed.

The present study has several limitations, including the relative small numbers of patients, which is counterbalanced by their homogeneity: all the patients had New York Heart Association (NYHA) class IV heart failure. The main limitation was the lack of a gold standard for measuring GFR, which made it impossible to determine which method is best for determining renal function among this population.

## CONCLUSION

We described the correlations and agreements using three equations to estimate GFR in a special population with advanced heart failure. Despite good correlations and agreements in comparing all three methods, the MDRD equation was more sensitive for identifying patients with GFR less than 60 ml/min than was either the CG or the new CKD-EPI formula.

## References

[1] Udani SM, Koyner JL (2010). The effects of heart failure on renal function. Cardiol Clin.

[2] Patel UD, Greiner MA, Fonarow GC (2010). Associations between worsening renal function and 30-day outcomes among Medicare beneficiaries hospitalized with heart failure. Am Heart J.

[3] Balfour IC, Srun SW, Wood EG (2006). Early renal benefit of rapamycin combined with reduced calcineurin inhibitor dose in pediatric heart transplantation patients. J Heart Lung Transplant.

[4] Bestetti R, Theodoropoulos TA, Burdmann EA (2006). Switch from calcineurin inhibitors to sirolimus-induced renal recovery in heart transplant recipients in the midterm follow-up. Transplantation.

[5] National Kidney Foundation (2002). K/DOQI clinical practice guidelines for chronic kidney disease: evaluation, classification, and stratification. Am J Kidney Dis.

[6] Levey AS, Bosch JP, Lewis JB (1999). A more accurate method to estimate glomerular filtration rate from serum creatinine: a new prediction equation. Modification of Diet in Renal Disease Study Group. Ann Intern Med.

[7] Cockcroft DW, Gault MH (1976). Prediction of creatinine clearance from serum creatinine. Nephron.

[8] Stevens LA, Schmid CH, Greene T (2010). Comparative performance of the CKD Epidemiology Collaboration (CKD-EPI) and the Modification of Diet in Renal Disease (MDRD) Study equations for estimating GFR levels above 60 mL/min/1 73 m2. Am J Kidney Dis.

[9] Levey AS, Stevens LA, Schmid CH (2009). A new equation to estimate glomerular filtration rate. Ann Intern Med.

[10] Scrutinio D, Passantino A, Santoro D, Catanzaro R (2011). The cardiorenal anaemia syndrome in systolic heart failure: prevalence, clinical correlates, and long-term survival. Eur J Heart Fail.

[11] Dries DL, Exner DV, Domanski MJ, Greenberg B, Stevenson LW (2000). The prognostic implications of renal insufficiency in asymptomatic and symptomatic patients with left ventricular systolic dysfunction. J Am Coll Cardiol.

[12] O'Meara E, Chong KS, Gardner RS (2006). The Modification of Diet in Renal Disease (MDRD) equations provide valid estimations of glomerular filtration rates in patients with advanced heart failure. Eur J Heart Fail.

[13] Camargo EG, Soares AA, Detanico AB (2011). The Chronic Kidney Disease Epidemiology Collaboration (CKD-EPI) equation is less accurate in patients with Type 2 diabetes when compared with healthy individuals. Diabet Med.

[14] Alper AB, Yi Y, Rahman M (2011). Performance of estimated glomerular filtration rate prediction equations in preeclamptic patients. Am J Perinatol.

[15] Corsonello A, Pedone C, Lattanzio F (2011). Chronic kidney disease and 1-year survival in elderly patients discharged from acute care hospitals: a comparison of three glomerular filtration rate equations. Nephrol Dial Transplant.

[16] Tinti F, Lai S, Umbro I (2010). Chronic kidney disease-epidemiology formula and model for end-stage liver disease score in the assessment of renal function in candidates for liver transplantation. Transplant Proc.

[17] Malyszko J, Przybylowski P, Malyszko JS (2010). Prevalence of chronic kidney disease in orthotopic heart transplant recipients and kidney allograft recipients using the new Chronic Kidney Disease Epidemiology Collaboration formula. Transplant Proc.

[18] Przybylowski P, Malyszko J, Malyszko J (2010). Chronic kidney disease in prevalent orthotopic heart transplant recipients using a new CKD-EPI formula. Ann Transplant.

[19] Gardner RS, Chong KS, O'Meara E (2007). Renal dysfunction, as measured by the modification of diet in renal disease equations, and outcome in patients with advanced heart failure. Eur Heart J.

[20] Scrutinio D, Passantino A, Catanzaro R, Guida P (2012). Clinical utility of different estimates of renal function for predicting mortality in chronic heart failure. Int J Cardiol.

